# Gut Microbiota Profile in Children with IgE-Mediated Cow’s Milk Allergy and Cow’s Milk Sensitization and Probiotic Intestinal Persistence Evaluation

**DOI:** 10.3390/ijms22041649

**Published:** 2021-02-06

**Authors:** Maurizio Mennini, Sofia Reddel, Federica Del Chierico, Simone Gardini, Andrea Quagliariello, Pamela Vernocchi, Rocco Luigi Valluzzi, Vincenzo Fierro, Carla Riccardi, Tania Napolitano, Alessandro Giovanni Fiocchi, Lorenza Putignani

**Affiliations:** 1 Translational Research in Pediatric Specialities Area, Allergy Unit, Bambino Gesù Children’s Hospital, IRCCS, 00147 Rome, Italy; maurizio.mennini@opbg.net (M.M.); roccoluigi.valluzzi@opbg.net (R.L.V.); vincenzo.fierro@opbg.net (V.F.); carla.riccardi@opbg.net (C.R.); tania.napolitano@opbg.net (T.N.); agiovanni.fiocchi@opbg.net (A.G.F.); 2 Multimodal Laboratory Medicine Research Area, Unit of Human Microbiome, Bambino Gesù Children’s Hospital, IRCCS, 00147 Rome, Italy; sofia.reddel@opbg.net (S.R.); federica.delchierico@opbg.net (F.D.C.); quagliariello.andrea@gmail.com (A.Q.); pamela.vernocchi@opbg.net (P.V.); 3 GenomeUp, Via Nemorense 91, 00199 Rome, Italy; simone@genomeup.com; 4 Department of Diagnostic and Laboratory Medicine, Unit of Parasitology and Multimodal Laboratory Medicine Research Area, Unit of Human Microbiome, Bambino Gesù Children’s Hospital, IRCCS, 00147 Rome, Italy

**Keywords:** cow’s milk allergic (CMA), cow’s milk sensitized (CMS), probiotics, quantitative real-time PCR assays, gut microbiota profiling

## Abstract

Food allergy (FA) and, in particular, IgE-mediated cow’s milk allergy is associated with compositional and functional changes of gut microbiota. In this study, we compared the gut microbiota of cow’s milk allergic (CMA) infants with that of cow’s milk sensitized (CMS) infants and Healthy controls. The effect of the intake of a mixture of *Bifidobacterium longum* subsp. *longum* BB536, *Bifidobacterium breve* M-16V and *Bifidobacterium longum* subsp. *infantis* M-63 on gut microbiota modulation of CMA infants and probiotic persistence was also investigated. Gut microbiota of CMA infants resulted to be characterized by a dysbiotic status with a prevalence of some bacteria as *Haemophilus*, *Klebsiella*, *Prevotella*, *Actinobacillus* and *Streptococcus*. Among the three strains administered, *B.* *longum* subsp. *infantis* colonized the gastrointestinal tract and persisted in the gut microbiota of infants with CMA for 60 days. This colonization was associated with perturbations of the gut microbiota, specifically with the increase of *Akkermansia* and *Ruminococcus*. Multi-strain probiotic formulations can be studied for their persistence in the intestine by monitoring specific bacterial probes persistence and exploiting microbiota profiling modulation before the evaluation of their therapeutic effects.

## 1. Introduction

Food allergy (FA) is a common chronic condition characterized by hypersensitivity reactions to a certain food, such as cow’s milk proteins [1].

The prevalence of IgE-mediated cow’s milk allergy (CMA) in infancy and early childhood has been estimated at 2–3% worldwide [2]. In Europe, a large cohort study confirmed challenge-proven cow’s milk allergy in <1% of children up to 2 years old [3]. Children with CMA in the first year of life carry an increased risk of being affected by subsequent other atopic diseases [4,5]. Therefore, understanding CMA etiology is needed for the management and prevention of the disease and its later impact on patients.

Several epidemiologic factors suggest that CMA may be linked to microbiota alterations. Among them, its association with C-section, low maternal intake of fermented foods during pregnancy, maternal dysbiosis, high maternal socioeconomic status, intrapartum antibiotic prophylaxis and the use of antibiotics in the nursery [6]. 

These observations generated the hypothesis that a lack of microbial exposure during infancy may be one of the responsible factors of cow’s milk allergy. According to the extended hygiene hypothesis, also known as the “microflora hypothesis of allergic disease” [7,8], the gut microbiota seems to be a key player in the early development of the host immune system [9]. Early microbial exposure moves the Th_1_/Th_2_ immune balance towards a Th_1_ phenotype [10,11] while a failure of the normal intestinal bacterial colonization is associated with a shift to a Th_2_ response, thus sustaining allergic manifestations [12,13]. Additionally, the establishment of oral tolerance to dietary antigens is, at least in part, dependent on the presence of commensal microbes [14,15]. Thus, it is not surprising that FA and particularly CMA have been associated with compositional and functional changes of gut microbiota [16,17].

Among gut microbiota phyla, Bacteroidetes, Proteobacteria, and Actinobacteria resulted significantly reduced in FA children, while Firmicutes was highly enriched. In particular, *Clostridium* and *Anaerobacter* have been identified as bacterial markers in these patients [16].

Furthermore, the gut microbiota dysbiosis, characterized by the enrichment of *Bacteroides* and *Alistipes,* has been described in non-IgE-mediated CMA [17].

In children with food sensitization, the increase of Enterobacteriaceae/Bacteroidaceae ratio and a decrease of Ruminococcaceae abundance have been described, suggesting that early gut dysbiosis contributes to subsequent development of FA [7]. 

To the best of our knowledge, only one study evaluated gut microbiota differences in food allergic versus food sensitized and healthy controls. Particularly, *Haemophilus*, *Dialister*, *Dorea* and *Clostridium* were decreased in subjects with food sensitization, while *Citrobacter*, *Oscillospira*, *Lactococcus* and *Dorea* were less abundant in subjects with FA [18].

On this premise, probiotics have gained widespread attention in recent years as a preventive and therapeutic option for FA. They may act as immune modulators that stimulate Th_1_-mediated responses. Their positive effects on FA symptoms are largely described in the literature [19].

The most common bacteria used as probiotics belong to *Lactobacillus* and *Bifidobacterium* species. Several studies found that *Bifidobacterium* spp. have anti-allergic properties in both animal models and clinical trials [20,21,22]. However, the timing and duration of probiotic treatment, the optimal probiotic strains and factors that may alter their action on the gut microbiota still need further research. Before proposing specific probiotic strains for clinical use it would be necessary to explore their ability to reach the intestinal lumen and to modify gut host microbiota [9].

This is one of the reasons why univocal recommendations about probiotics have not been yet drawn up for FA. A multitude of administration approaches has been proposed for CMA, investigating the effect of different doses and intake timing [22].

In the present study, we aimed to describe the gut microbiota ecology and composition of CMA infants, cow’s milk sensitized (CMS) infants and healthy controls. In this context, we also evaluated the persistence of a probiotic mix in the gastrointestinal tract of CMA infants and its impact on gut microbiota modulation.

## 2. Results

### 2.1. Demographic and Clinical Profiling of Subjects

During the study period of 3-years, 124 patients aged 10–15 months were evaluated for suspected CMA at our hospital units. Ninety–seven patients were excluded: 14 were treated with proton-pump inhibitors (PPI), 22 were still partially breastfed, 38 had taken a course of antibiotic therapy in the previous 6 months, 52 had probiotics and 55 had functional foods in the last 2 months; 67 infants had more than one reason for exclusion. The main reasons for exclusion were the use of probiotics and feeding an extensively hydrolysed cow’s milk formula with added functional foods (probiotics or prebiotics), prescribed by the paediatrician to replace breast milk (52 cases). 

The study was proposed to 27 children. As one of them was diagnosed with celiac disease soon after the double-blind placebo-controlled food challenge (DBPCFC) he was excluded, hence 26 children were enrolled (median age 12.8 months).

The CMA group included 14 infants, the CMS group 12 DBPCFC-negative infants, and the Healthy group 14 age- and sex-matched healthy infants (Table 1). There was no significant difference between groups in anthropometric characteristics including sex, age, mode of delivery, duration of breastfeeding, and duration of cow’s milk-free diet (Table 1).

Three out of 14 (21.4%) in the CMA group, 3/12 (25%) in the CMS group and 6/14 (43%) in the Healthy group were delivered by caesarean section (*p* = 0.6). Thirty-eight infants had been breastfed for more than two months, exclusively or complementarily (Table 1). 

Skin prick test with fresh cow‘s milk returned positive in 11/14, 6/12, 0/14 in CMA, CMS and Healthy groups, respectively. Specific IgE for cow’s milk was positive in 13/14 and 9/12, in CMA and CMS groups respectively.

After DBPCFC, all CMA infants followed a cow’s milk protein’s free diet and regularly took the probiotic mixture twice daily for 30 days without any relevant adverse events. 

### 2.2. RT-PCR Analysis

**Figure 1:**
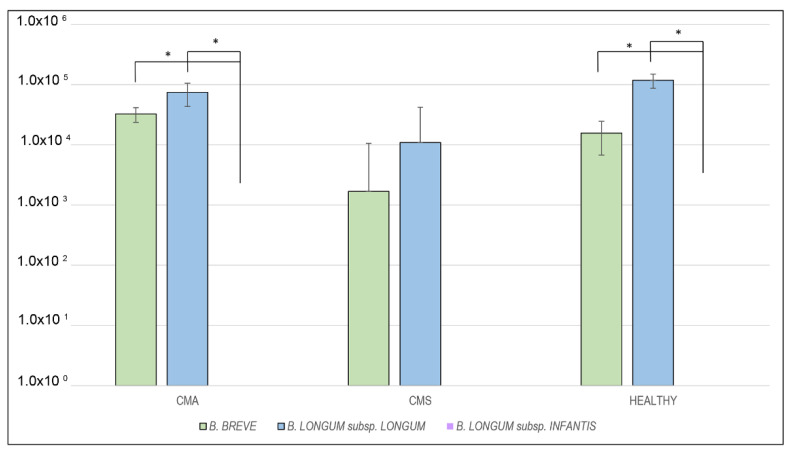
Median differences between *B. breve*, *B. longum* subsp. *longum* and *B. longum* subsp. *infantis* levels expressed as molecules/ul at T_0_ for the three analyzed groups (* *p* < 0.05).

At baseline, significant differences between *B. breve* and *B. infantis* and between *B. longum* and *B. infantis* were found for CMA and Healthy groups, respectively (Figure 1, Supplementary Table S2). 

*B. breve* and *B. longum* were present in the gut microbiota of the three groups in the order of 10^3^–10^5^ molecules/ul (Figure 1), without significant differences (Supplementary Table S3). No trace of *B. longum* subsp. *infantis* was detected in the three groups. 

**Figure 2:**
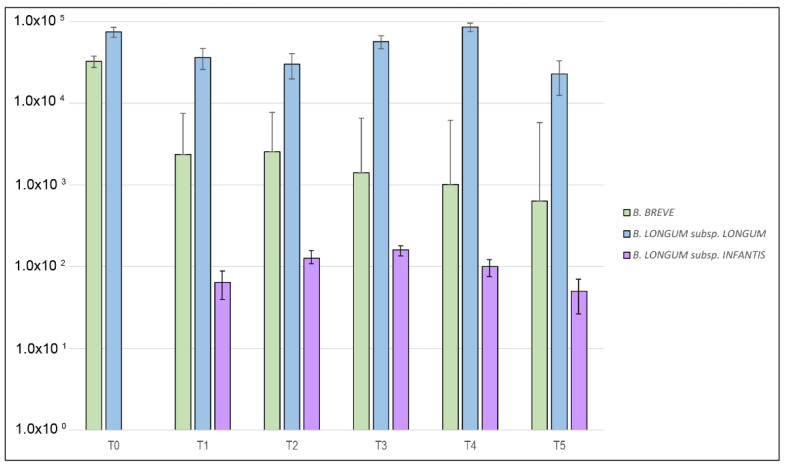
Median differences between *B. breve*, *B. longum* subsp. *longum* and *B. longum* subsp. *infantis* levels expressed as molecules/ul at each point of the time-course for the cow’s milk allergic (CMA) group.

During the probiotic intervention, no significant variations of *B. breve* and *B. longum* subsp. *longum* amounts were detected in the CMA group, while the amount of *B. longum* subsp. *infantis,* significantly increased from T_1_ to T_3_ and decreased since point T_4_ (Figure 2, Supplementary Table S4). 

### 2.3. Gut Microbiota Profiling

**Figure 3:**
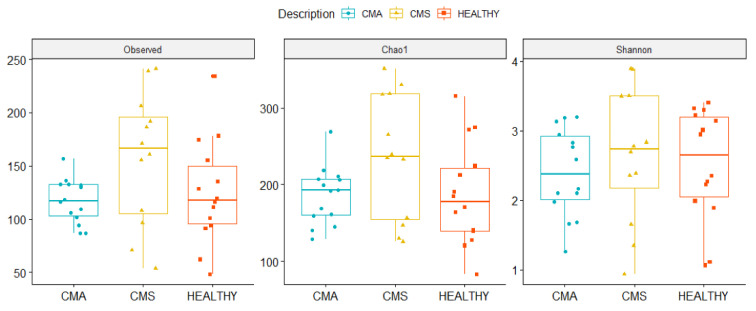
Boxplots representing alpha-diversity computed by Observed, Chao1 and Shannon indexes in the three CMA, cow’s milk sensitized (CMS), and HEALTHY groups. Boxes represent the median, 25th and 75th percentiles calculated for CMA (blue), CMS (yellow), and HEALTHY (red) groups.

Alpha diversity was calculated using Observed, Chao1 and Shannon indices in order to evaluate Operational Taxonomic Units (OTUs) *evenness, rarity* and *richness* for CMA, CMS, and Healthy groups, at baseline. No statistically significant differences were found in the three indices comparisons. However, CMS showed the highest number of total and rare OTUs (i.e., Observed and Chao I index, respectively), while CMA was characterized by the lowest OTUs richness (i.e., Shannon index) (Figure 3). 

Beta diversity was computed to estimate the phylogenetic relatedness (i.e., unweighted UniFrac algorithm) of CMA, CMS and Healthy groups. CMA group was clearly separated from the other groups, as verified by the PERMANOVA test (*p* = 0.019) (Supplementary Figure S1).

**Figure 4:**
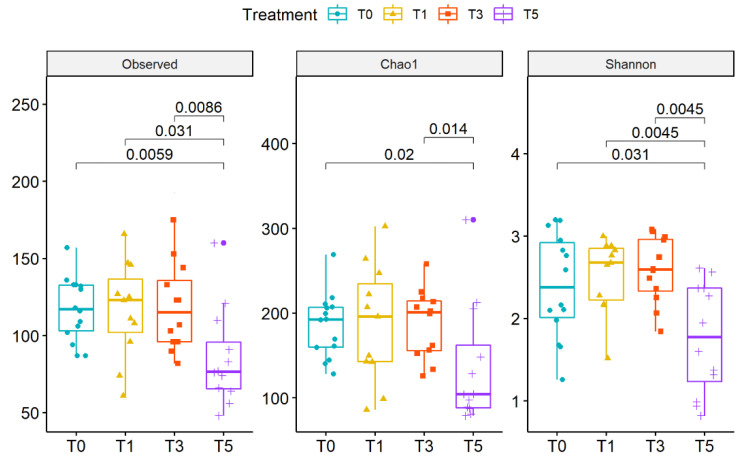
Boxplots representing alpha-diversity by Observed, Chao1 and Shannon indexes during the time-course for the CMA group. Boxes represent the median, 25th and 75th percentiles calculated for CMA group at T_0_ (blue), T_1_ (yellow), T_3_ (red) and T_5_ (purple). *p*-values are reported.

After probiotics intake, no statistically significant changes of alpha diversity were observed in the CMA group. At T_5_ a statistically significant decrease was observed for all alpha diversity indices (Figure 4). 

No clearly defined patient clusters along the entire time-course was detected (PERMANOVA = 0.829), (Supplementary Figure S2).

To detect differences in OTU composition, we compared the three groups, at phylum and genus levels. 

At the phylum level, Verrucomicrobia was highest in Healthy and gradually decreased from CMS to CMA group (*p* < 0.05) (Supplementary Table S5). On the contrary, Firmicutes resulted more abundant in the CMS group and lowest in the healthy subjects (Supplementary Figure S3).

**Figure 5:**
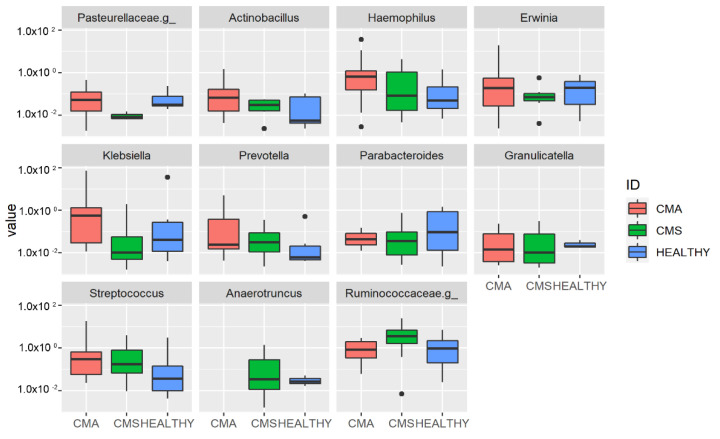
Operational Taxonomic Unit (OUT) distribution of the gut microbiota for the three groups’ comparison assessed by DeSeq2 test at genus level. The interquartile range is represented by the box and the line in the box is the median. The whiskers indicate the largest and the lowest data points, respectively, while the dots symbolize outliers. Only statistically significant OTUs (*p* < 0.05) are plotted.

At the genus level, *Haemophilus*, *Actinobacillus*, *Prevotella*, *Klebsiella* and *Streptococcus* were associated with the CMA group (*p* < 0.05, Supplementary Table S6) while *Parabacteroides* and *Granulicatella* were more abundant in the Healthy group (Figure 5).

**Figure 6:**
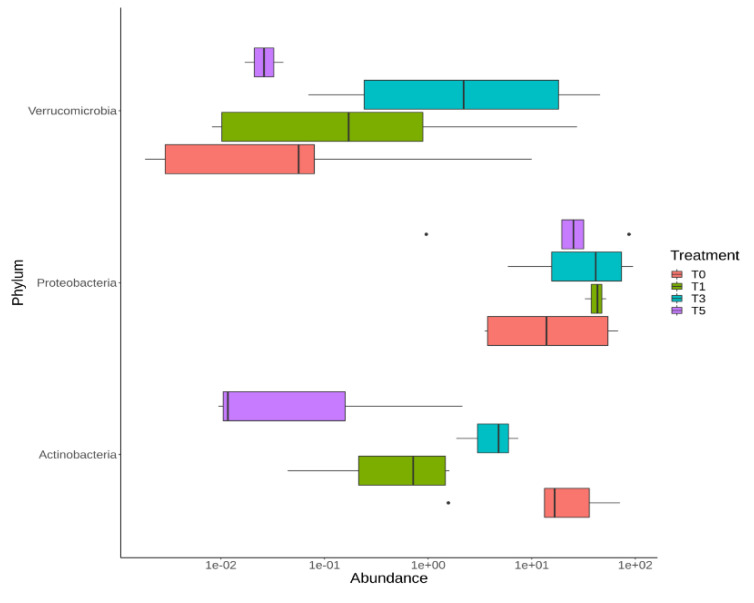
OTU distribution of the gut microbiota during the time-course of the CMA group computed by DeSeq2 test at phylum level. The interquartile range is represented by the box and the line in the box is the median. The whiskers indicate the largest and the lowest data points, respectively, while the dots symbolize outliers. Only statistically significant OTUs (*p* < 0.05) are plotted.

To evaluate the influence of probiotic intake on gut microbiota modulation, we tested the OTU distribution during the T_0_–T_5_ time-course in the CMA group. At the phylum level, there was an increase of Verrucomicrobia during probiotic intake with a peak of abundance at T_3_ and a rapid decrease at T_5_. Proteobacteria showed a gradual increase during probiotic intake and this increment was maintained also at T_5_. On the contrary, Actinobacteria decreased during the time-course, even if an increase was registered from point T_1_ to point T_3_ (Figure 6, Supplementary Table S7).

**Figure 7:**
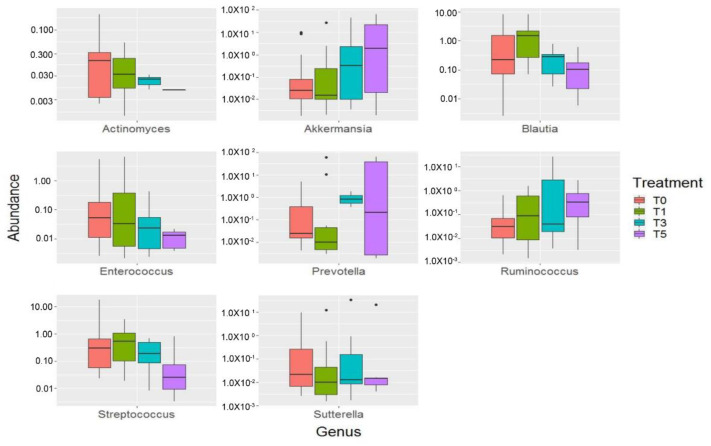
OTU distribution of the gut microbiota during the time-course of the CMA group computed by DeSeq2 test at the genus level. The interquartile range is represented by the box and the line in the box is the median. The whiskers indicate the largest and the lowest data points, respectively, while the dots symbolize outliers. Only statistically significant OTUs (*p* < 0.05) are plotted.

At the genus level, probiotic intake determined an increase of *Akkermansia*, *Prevotella* and *Ruminococcus* that was maintained also at T_5_. *Blautia* increased during the entire period of probiotic intake, until T_3_ point, while at T_5_ it started to decrease showing a level of abundance lower than that observed at T_0_. On the opposite way, *Actinomyces*, *Enterococcus*, *Streptococcus* and *Sutterella* resulted diminished after probiotic intervention (Figure 7, Supplementary Table S8).

## 3. Discussion

Several comparative studies have been conducted on the intestinal microbiota composition of healthy versus allergic children, but their results are controversial [7,8,23,24,25]. To the best of our knowledge only one study evaluated gut microbiota differences in food-allergic versus food sensitized and healthy controls [18]. 

We observed a lower microbial diversity in CMA infants, both in terms of richness and evenness indicating that bacterial OTUs are not equally abundant in the gut ecosystem, hence generating a disequilibrium or compositional dysbiosis. Indeed, previous studies found low intestinal diversity in CMA patients [26], supporting the theory of “microbial deprivation syndromes of affluence” [27]. According to this theory, the reduced microbial stimulation could lead to abnormal immune development in early life, a possible contributory cause of allergic disease [28]. This is in line with other previous results obtained for atopy and food allergy [29,30,31].

In our study harmful bacteria as *Haemophilus* and *Klebsiella*, *Prevotella* were associated with CMA.

Prior studies investigating the association of gut microbiota and allergic disease during infancy demonstrated inverse associations of *Haemophilus* with eczema [32] and food sensitization [18]. In vitro studies demonstrated that the pro-inflammatory activity of this bacteria leads to activation of inflammatory Th_2_ pathways suggesting a possible role in allergy manifestation [33]. 

Additionally, *Klebsiella* spp. have been associated with pro-inflammatory responses [34,35]. Moreover, a higher abundance of this genus has been correlated with atopy in infants [23]. So, our results confirm its possible association to later development of pediatric allergy [36,37]. 

Interestingly, *Haemophilus*, *Prevotella*, *Actinobacillus* and *Streptococcus* gradually decreased in the gut microbiota from CMA to CMS to Healthy infants, leading to suppose a correlation of these bacteria to allergic status. 

Probiotics have been proposed as a strategy for the management of CMA allergy. However, not all the studies based on probiotic intake demonstrate the actual ability of probiotics to colonize the GT. This makes it difficult to decide which strain and at what doses to administer it.

For this reason, we investigated the persistence of TRIBIF® probiotic strains in the gut microbiota at defined time-points. The mixture we studied is reported to get a significant improvement of symptoms and quality of life in allergic rhinitis and intermittent asthma [38], but no data have yet been provided about the real presence of probiotics in patients’ microbiota. 

From our study, *B. breve* and *B. longum* subsp. *longum* are present in the gut microbiota without significant differences in CMA, CMS and Healthy groups. *B. breve* and *B. longum* subsp. *longum,* commonly present in the gut of breastfed infants, are able to metabolize certain human milk oligosaccharides (HMOs) and carbohydrates released by other bifidobacteria. Moreover, *B. longum* subsp. *longum* persist in the gut microbiota also after weaning, due to its ability to utilize such diet-derived carbohydrates [39]. Our data confirm that they persist after weaning, and the probiotic supplementation has not produced any evaluable effect on the concentration of these species, which were already abundant in the infant gut microbiota. By contrast, *B. longum* subsp. *infantis* was totally absent at baseline in all groups. During probiotic intake, its amount increased significantly during the first 30 days, while it decreased after probiotic discontinuation. This indicates the survival of the bacteria during their transit in the gastrointestinal tract and their ability to colonize and to persist at least for some time in the gut microbiota of children affected by IgE-mediated CMA.

*B. longum* subsp. *infantis* is unique among gut microbes for its capacity to transport into its cytoplasm and to consume the full range of HMOs [40]. Then, it is presumable that *B. infantis* is a very early colonizer of the infant gut during breastfeeding, whereas after weaning this species is replaced by others more adaptable to the new environment. This may be the cause of the absence of *B. infantis* in all the groups at baseline.

It is known that the prolonged colonization of this species is a rare phenomenon [41], while both probiotic inoculation and continued provision of HMOs are likely to foster long-term colonization [42]. Our results are in agreement with these studies, indicating that between 10 and 15 months the colonization of *B. infantis* persists only if there is a continuous intake.

A beneficial impact of probiotic intake on gut microbiota modulation could be associated with the significant increase of *Akkermansia* that was also maintained after probiotic suspension. *A. muciniphila* is involved in the maintenance of intestinal barrier functions and in the containment of intestinal inflammation, playing a pivotal role in the host’s overall health status [43]. 

Additionally, the persisting reduction of *Streptococcus* after the probiotic supplementation [44] can be considered a positive effect of the intervention, as *Streptococcus* is a potentially pathogenic microorganism that could be directly involved in atopy [45] especially considering that it resulted in a biomarker of CMA in the present study.

*Blautia* distribution increased during all the period of probiotic intake, until T_3_ point, while at T_5_ it started to decrease, suggesting a possible synergistic mechanism with the probiotic species for gut microbiota colonization.

Additionally, *Ruminoccocus* increased during the probiotic intake and after probiotic suspension. Interestingly, low relative abundance of Ruminococcaceae were registered at one week of age in infants that developed IgE-associated eczema, and low abundance of the genus *Ruminococcus* at that age was associated with exaggerated Toll-Like Receptor (TLR)-2-induced interleukin (IL)-6 and Tumor necrosis factor (TNF)-α responses at 6 months [46]. The increase of *Ruminococcus* during and after probiotic intake may be beneficial also considering its ability to produce ruminococcins, such as ruminococcin A, a bacteriocin that can inhibit the development of *Clostridium* species [47].

Our results indicate that *B. infantis* could have the biological plausibility for therapeutic actions for CMA infants, while the two other strains administered probably do not. As multi-strain probiotic formulations are largely marketed, we suggest that, according to the 5th Bradford Hill criterion [48], an evaluation of the colonization and persistence of their specific probiotics in the intestine should precede clinical studies. 

This study has few limitations. It is not an efficacy study: we chose don’t include any clinical evaluation of the trend of milk allergy symptoms, because we had no indication of the kinetics of the probiotic. Moreover, this probiotic formulation has not been described in efficacy studies in allergic children. 

Another limitation is the small number of patients involved. However, we performed this study on a sample of children with homogeneous feeding, in a strict range of age which guarantees the uniformity stability of the gut microbiota composition. 

## 4. Materials and Methods

### 4.1. Patients Enrolment

This was a prospective multicentric study. Infants aged 10–15 months, evaluated for suspected IgE-mediated CMA, were consecutively enrolled at the Allergy Division of Bambino Gesù Children’s Hospital in Rome between January 2016 and December 2018. Infants who were still breastfed, those with gastrointestinal disease in the last 30 days, metabolic or congenital disorders were not included. We excluded infants who had been administered with antibiotics in the last 6 months, or probiotics, proton-pump inhibitors [49], fermented milk and other functional foods in the 4 weeks prior to the onset of the study. 

For each patient, informed consent was obtained from their parents, and anamnestic data were recorded. The sensitivity to cow’s milk was documented by a positive skin prick test (cut-off 3 mm wheel diameter) and/or allergen-specific IgE (ImmunoCAP^®^ Thermo Fisher Scientific, Uppsala, Sweden) with 0.35 kU/L as a lower limit of eligibility, as described elsewhere [50]. 

In positive patients, DBPCFC was then performed. 

### 4.2. Food Challenges 

Cow’s milk was administered in seven incremental doses of 0.1, 0.3, 1, 3, 10, 30, and 100 mL at 30 min intervals for three and a half hours. Neocate^®^ was used as a placebo and the milk without lactose was blinded according to the standardized AAAAI/Europrevall protocol [51,52]. Oral food challenge was discontinued at the first onset of objective symptoms [53]. Patients were observed up to 6 h after starting the test.

### 4.3. Study Design and Faecal Sample Collection

The included patients were divided into two groups:

CMA: patients sensitized to cow’s milk proteins with DBPCFC-confirmed IgE-mediated cow’s milk allergy;

CMS: sensitized but tolerant to cow’s milk proteins patients (negative DBPCFC).

A group of age- and sex-matched children without suspect of CMA, regularly formula-fed and subjected to the same exclusion criteria, was recruited at our immunization clinic (Healthy group). 

For each subject (CMA, CMS, Healthy groups), a stool sample was collected at enrolment and stored at −80°C at the Human Microbiome Unit of Bambino Gesù Children’s Hospital in Rome until further processing.

Patients belonging to the CMA group were treated with a probiotic mixture (*Bifidobacterium breve* M-16V, *Bifidobacterium longum* subsp. *longum* BB536 and *Bifidobacterium longum* subsp. *infantis* M-63; TRIBIF^®^, Valeas SpA, Milan, Italy). A dose of 3.5 × 10^9^ colony forming units (CFU)/dose of each species was administered twice per day for 30 consecutive days. Fecal samples were collected during clinical visits at time T_0_ (no probiotic intake), T_1_ (7 days of probiotic intake), T_2_ (15 days of probiotic intake), T_3_ (30 days of probiotic intake), T_4_ (after 60 days from probiotic discontinuation), T_5_ (after 90 days after probiotic discontinuation). All samples were stored at −80 °C at the Human Microbiome Unit of Bambino Gesù Children’s Hospital in Rome until further processing. 

After the oral challenge, all the positive children were prescribed a milk-free diet with an extensively hydrolysed whey formula not containing prebiotics or probiotics (Aptamil SP, Danone-Nutricia, Utrecht NL, The Netherlands). Healthy controls continued to take their regular formula, while CMS children were allowed a free diet with the substitution of a formula not enriched with functional foods (Mio 2, Nestlé Vevey, Switzerland). 

This study, designed according to the Declaration of Helsinki, was registered on Clinicaltrials.gov (ClinicalTrials.gov Identifier: NCT03639337; 21 August 2018), and was approved by Bambino Gesù Children’s Hospital IRCCS Ethics Committee (protocol number 787_OPBG_2014). 

### 4.4. Isolation of B. breve M-16V, B. longum subsp. longum BB536 and B. longum subsp. Infantis M-63, Bacterial DNA Extraction and ITS Locus Amplification

*B. breve* M-16V, *B. longum* subsp. *longum* BB536 and *B. longum* subsp. *infantis* M-63 were cultured, isolated and confirmed for identification as already described [54]. DNA was automatically extracted from bacterial colonies using biorobot EZ1 following the manufacturer’s instructions (Qiagen, Hilden, Germany). The entire ITS (internal transcription spacer) locus (1430 bp) was amplified using universal primers (FW: 5′-GGT GTG AAA GTC CAT CGC T-3′; RV: 5′-GTC TGC CAA GGC ATC CAC CA-3′). PCR was performed in a reaction mix containing 5 µL 10× Buffer, 2 µL 2.5 mM MgCl_2_, 1 µL each primer (10 µmol/L), 2 µL dNTPs (10 mmol/L), 1 µL Taq DNA polymerase (5 U/µL) (KAPA Taq PCR kit, KAPA Biosystems, Boston MA, USA), 5 µL DNA template (10 ng/µL) and molecular-grade H_2_O to a final reaction volume of 50 µL. The amplification protocol consisted of one cycle of initial denaturation at 94 °C for 5 min, 30 cycles of denaturation at 94 °C for 1 min, annealing at 56.5 °C for 1 min, and extension at 72 °C for 1 min followed by a final extension at 72 °C for 10 min. Amplicons were purified using centrifugal filter units (Amicon Ultra-0.5 mL Centrifugal filters 30 K, Sigma-Aldrich, St. Louis, MO, USA) and quantified using the NanoDrop ND-1000 spectrophotometer (Thermo Fisher Scientific, Waltham, MA, USA).

### 4.5. Cloning and RT-PCR Assay Design 

The pGEM^®^-T Easy Vector System kit (Promega, Milan, Italy) was used to clone the purified PCR fragment into the PGEM vector with the use of *Escherichia coli* competent cells. The extraction of recombinant plasmids pGEM-BB (pGEM+*B. breve*), pGEM-BL (pGEM+*B. longum* subsp. *longum*) and pGEM-BI (pGEM+*B. longum* subsp. *infantis*) was then performed (Plasmid Miniprep Kit, Promega, Italy). Obtained DNA was quantified using the NanoDrop ND-1000 spectrophotometer and serial dilutions of plasmids were prepared in the range from 10^6^ to 10^1^ vector copy numbers and used to create a standard curve for the quantitative RT-PCR (qRT-PCR) assays. ITS rDNA cloned fragments were amplified and sequenced (Genetic Analyser 3500, Applied Biosystems, Foster City, CA, USA) according to the manufacturer’s instructions. FASTA sequences were aligned with CLUSTAL-W software (http://www.ebi.ac.uk/clustalw/) (accessed on 1 February 2021) and species-specific primers and TaqMan probes (Roche Diagnostics) design was carried out (Supplementary Table S1).

### 4.6. Stool Samples DNA Extraction

DNA extraction was executed on 200 mg of stool sample by the use of QIAmp Fast DNA Stool mini kit (Qiagen). The obtained DNA was quantified by NanoDrop ND-1000 and roughly diluted to 16 ng/µL for a total amount of 80 ng per assay.

### 4.7. qRT-PCR Assay

The amounts of *B. breve*, *B. longum* subsp. *longum* and *B. longum* subsp. *infantis* in faecal samples were measured by qRT-PCR in the Light Cycler 480 instrument (Roche Diagnostics, Mannheim, Germany) and following the procedure already described in detail [54].

### 4.8. 16S rRNA Targeted-Metagenomics

The 16S rRNA targeted-metagenomics was performed on T_0_ samples of CMA, CMS, and Healthy groups and on the follow-up samples of the CMA group (T_1_, T_3_ and T_5_ time-points).

The variable region V3-V4 of the 16S rRNA gene (∼460 bp) was amplified by using the primer pairs 16S_F 5′ -(TCG TCG GCA GCG TCA GAT GTG TAT AAG AGA CAG CCT ACG GGN GGC WGC AG)-3′ and 16S_R 5′ -(GTC TCG TGG GCT CGG AGA TGT GTA TAA GAG ACA GGA CTA CHV GGG TAT CTA ATC C)-3′ reported in the 16S Metagenomic Sequencing Library Preparation protocol (Illumina, San Diego, CA, USA). The first PCR reaction was set up using Fast Start Hifi Taq (Roche Diagnostics) with the following conditions: initial denaturation at 95 °C for 3 min, 32 cycles of denaturation at 95 °C for 30 s, annealing at 55 °C for 30 s, and extension at 72 °C for 30 s, and a final extension step at 72 °C for 5 min.

Twenty ul of KAPA Pure Beads (Roche Diagnostics) were employed to purify DNA amplicons. To obtain a unique combination of bar-code sequences, a second amplification step was prepared using Nextera technology (Illumina). The final library was cleaned-up using 50 μL of KAPA Pure Beads, quantified using Quant-iT™ PicoGreen^®^ dsDNA Assay Kit (Thermo Fisher Scientific) and diluted in equimolar concentrations (4 nM). The following steps consisted of samples’ pooling, denaturation and dilution to 7 pM. Samples were then sequenced on an Illumina MiSeqTM platform (Illumina) following the manufacturer’s specifications to generate paired-end reads of 250 base-length.

### 4.9. Biocomputational and Statistical Analyses

Illumina Miseq reads were first analyzed for their quality, length and chimera presence using the Qiime v1.8 pipeline. Then, sequences were organized into Operational Taxonomic Units (OTUs) with a 97% of clustering threshold of pairwise identity. PyNAST v.0.1 program was used to carry out a multiple sequence alignment against Greengenes 13_08 database with a 97% similarity for bacterial sequences [55]. The OTU multiple sequence alignment was used as well to build a phylogenetic tree [56]. In order to filter rarely distributed OTUs, and avoid noise (i.e., artifact taxa), taxa with a threshold of prevalence greater than 0.05% within the whole dataset were selected. Alpha and beta diversity were performed by *phyloseq* of R package [57] and PERMANOVA test was applied on beta diversity metrics with 9999 permutations to compare samples at different time points. With the aim to identify microbial biomarkers, differences in the relative abundances of taxa between groups and during the time-course of the CMA group was assessed by DESeq2 test at phylum and genera taxonomic level. In particular, for the genus level, only taxa with a relative abundance higher than 0.01 were considered for the computational analysis [58]. SPSS statistical software (version 21) was used for graphical analysis of probiotic species persistence during the time-course and to perform Wilcoxon signed rank test for paired comparison between time-points (Supplementary Tables S2 and S3). Student’s t-test was applied for comparison of clinical variables of the population.

### 4.10. Metagenomic Data Open Access Repository

All raw sequencing reads are available at NCBI BioProject database (PRJNA644788) (https://www.ncbi.nlm.nih.gov/bioproject/) (accessed on 1 February 2021).

## 5. Conclusions

In conclusion, our study revealed that the gut microbiota of children with CMA is characterized by the prevalence of harmful bacteria such as *Haemophilus*, *Klebsiella* and *Prevotella* which can be considered as possible biomarkers associated with the disease. 

Moreover, we can confirm the ability of *B. longum* subsp. *infantis* to pass the gastrointestinal tract and to persist in the gut microbiota of infants with CMA. 

Potentially beneficial modulation of the gut microbiota during the probiotic intake was also observed and was maintained for some genera as *Akkermansia* and *Ruminococcus*.

Considering the protective and anti-inflammatory properties of *B. infantis* [40], it would be interesting to investigate, in a clinical trial, its clinical effect on food allergy symptoms. 

Moreover, it would be interesting in a future study to evaluate the gut microbiota profile in children with a history of allergy with a negative oral challenge and sensitized at the enrollment, and compare that data with our results on sensitized children in order to investigate common fingerprints in terms of gut microbiota composition.

## Figures and Tables

**Table 1:** Demographic and clinical characteristics of subjects. N/A, not applicable.

Features	CMA	CMS	Healthy	*p*-Value
Mean age ± SD (months)	12.9 ± 1.7	13.4 ± 1.0	12.6 ± 1.5	0.08
Male/Female	8/6	7/5	6/8	0.70
Cesarean section (%)	21%	25%	43%	0.60
Mean breastfeeding duration (months)	7.2	7.5	8	0.44
Mean cow’s milk free diet duration ± SD (months)	6.3 ± 4.1	8.1 ± 4.6	N/A	0.51
Mean diameter Prick by Prick with Cow’s Milk (mm)	5.0	1.3	0.0	0.09
Mean diameter Skin Prick Test with Alpha-Lactalbumin (mm)	4.4	1.0	0.0	0.04
Mean diameter Skin Prick Test with Beta-Lactoglobulin (mm)	3.8	0.9	0.0	0.20
Mean diameter Skin Prick Test with Casein (mm)	6.4	1.0	0.0	0.04
Total IgE (kU/L)	228.2	51.2	N/A	<0.01
Specific IgE for Cow’s milk (kUA/L)	23.8	0.8	N/A	0.05
Specific IgE for α-Lactalbumin (kUA/L)	3.5	0.1	N/A	0.14
Specific IgE for β-Lactoglobulin (kUA/L)	9.0	0.5	N/A	0.26
Specific IgE for Casein (kUA/L)	9.4	0.4	N/A	0.18

## Data Availability

All raw sequencing reads are available at NCBI BioProject database (PRJNA644788) (https://www.ncbi.nlm.nih.gov/bioproject/) (accessed on 1 February 2021).

## Supplementary Materials

The following are available online at https://www.mdpi.com/1422-0067/22/4/1649/s1.
