# Supplementary material for: Gut Microbiota Profile in Children with IgE-Mediated Cow’s Milk Allergy and Cow’s Milk Sensitization and Probiotic Intestinal Persistence Evaluation
Source: Int J Mol Sci. 2021 Feb 6;22(4):1649. doi: 10.3390/ijms22041649 (PMC7915344; doi:10.3390/ijms22041649)
Supplement: Supplementary file 1 [file ijms-22-01649-s001.pdf]

Supplementary Materials

The supplementary Materials contain: 3 Supplementary Figures, 8 Supplementary Tables (Supplementary Tables 1–8).

Supplementary Figures

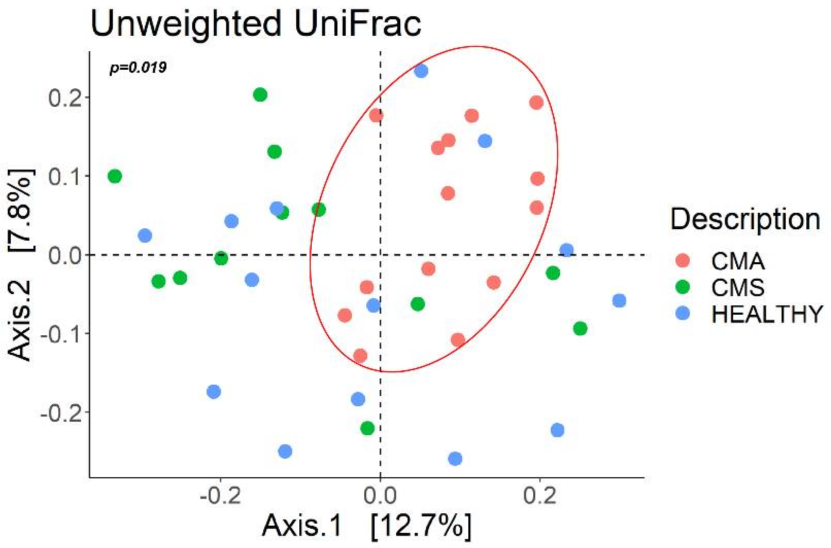

Supplementary Figure S1. Beta-diversity analysis applied for CMA, CMS and Healthy samples. The plots show the first two principal axes for PCoA using unweighted UniFrac algorithm. p-value obtained by PERMANOVA analyses is reported.

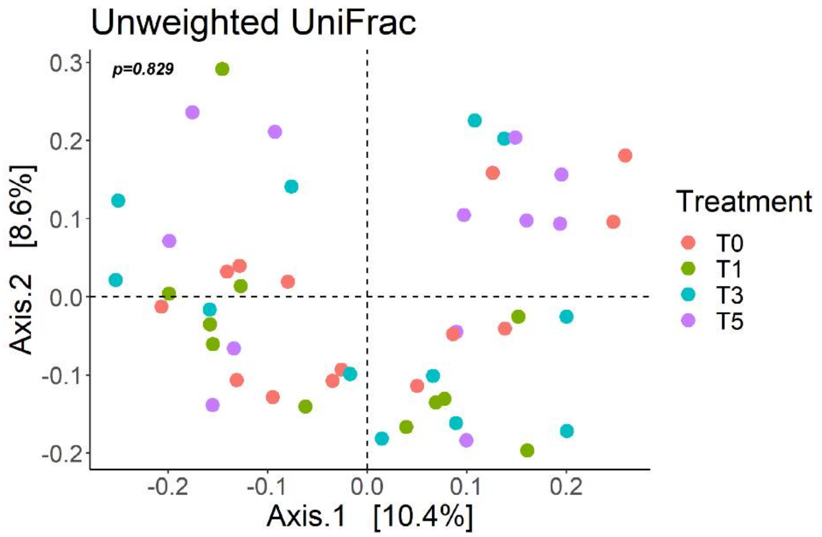

Supplementary Figure S2. Beta-diversity analysis applied for all the time points of the CMA group. The plots show the first two principal axes for PCoA using unweighted UniFrac algorithm. *p*-value obtained by PERMANOVA analyses is reported.

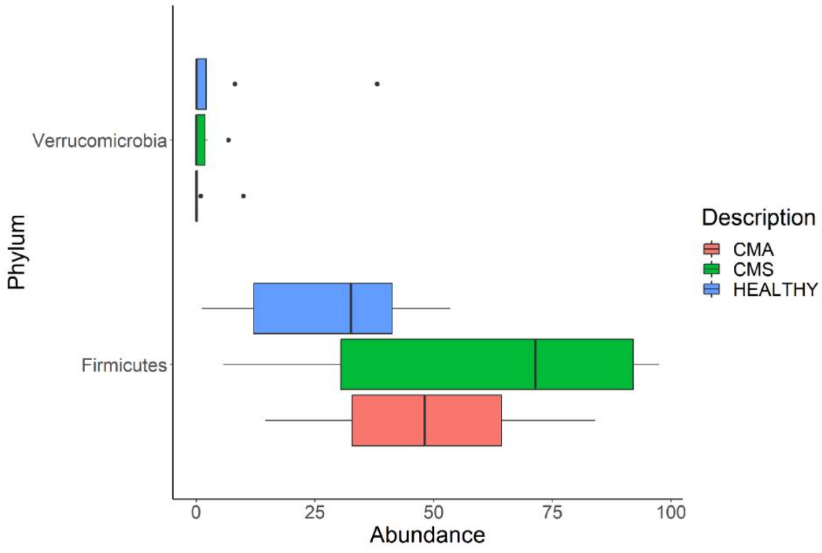

Supplementary Figure S3. OTU distribution of the gut microbiota for the 3 groups' comparison assessed by DeSeq2 test at phylum level. The interquartile range is represented by the box and the line in the box is the median. The whiskers indicate the largest and the lowest data points, respectively, while the dots symbolize outliers. Only statistically significant OTUs (p<0.05) are plotted.

Supplementary Tables.

Supplementary Table S1. Primers and probes designed for *B. breve*, B. *longum* subsp. *longum* and B. *longum* subsp. *infantis* for the Real Time assays.

Species specificity	Primer or probe	Sequence	Product size
B. breve	Fw_Bbreve	5′-gTg gTg gCT TgA gAA CTg gAT Ag 3′	117 bp
	Rv_Bbreve	5′-CAA AAC gAT CgA AAC AAA CAC TAA A-3′	
	P_Bbreve	5′-FAM-CGC ACC CAC CGC A-BHQ-3′	
B. longum subsp. *longum*	Fw_*Blongum* subsp. *longum*	5′-Tgg AAg ACg TCg TTg gCT TT-3'	109 bp
	Rv_ *Blongum* subsp. *longum*	5′-ATC gCg CCA ggC AAA A-3′	
	P_ *Blongum* subsp. *longum*	5′-FAM-CGC ACC CAC CGC A-BHQ-3′	
B. longum subsp. *infantis*	Fw_*Blongum* subsp. *infantis*	5′-CTC CTT TCT ACg gAg AAT ACA ggA T-3′	113 bp
	Rv_ *Blongum* subsp. *infantis*	5′-gAA CCg AAA gCC CAT gAT CT-3′	
	P_ *Blongum* subsp. *infantis*	5′-CgC gAT ggT CgC gTg Tgg A-3′	

Supplementary Table S2. Wilcoxon Signed Rank Test applied for *B. breve*, *B. longum* and *B. infantis* amount comparisons within each of the three CMA, CMS, HEALTHY groups.

Probiotic Species	CMA	CMS	HEALTHY
Paired comparison		*p*-value [1]	
B. breve vs *B. longum*	0.311	0.600	0.055
B. breve vs *B. infantis*	**0.004**	0.138	**0.006**
B. longum vs *B. infantis*	**0.003**	0.068	**0.001**

[1] values in bold are statistically significant.

Supplementary Table S3. Wilcoxon Signed Ranks Test applied for *B. breve*, *B.longum* and *B. infantis* amount between CMA, CMS, and HEALTHY group paired comparisons.

B. breve	*B. longum*	*B. infantis*

Group	*p*-value	Group	*p*-value	Group	*p*-value
CMA vs CMS	0.116	CMA *vs* CMS	0.463	CMA vs CMS	0.285
CMA vs HEALTHY	0.279	CMA vs HEALTHY	0.753	CMA vs HEALTHY	0.500
CMS vs HEALTHY	0.686	CMS vs HEALTHY	0.463	CMS vs HEALTHY	1

Supplementary Table S4. Wilcoxon Signed Ranks Test applied for the time point comparisons of *B. breve, B. longum* and *B. infantis* values in the CMA group.

Groups	*B. breve*	*B. longum* subsp. *longum*	*B. longum* subsp. *infantis*
		p value	
T1 - T0	0.480	0.433	**0.003**
T2 - T0	0.433	0.272	**0.01**
T3 - T0	0.091	0.182	**0.005**
T4 - T0	**0.019**	0.875	**0.016**
T5 - T0	**0.008**	0.477	0.173
T2 - T1	0.433	0.875	0.084
T3 - T1	0.213	0.929	0.093
T4 - T1	0.583	0.209	0.48
T5 - T1	**0.010**	0.477	0.374
T3 - T2	0.182	1.000	0.248
T4 - T2	0.814	0.182	0.388
T5 - T2	**0.008**	0.859	0.131
T4 - T3	0.722	0.062	0.721
T5 - T3	0.139	0.959	0.374
T5 - T4	**0.016**	**0.037**	0.799

values in bold are statistically significant.

Supplementary Table S5. DeSeq2 test statistically significant results for CMA, CMS and HEALTHY group comparison at phylum level. For each group mean relative abundances of phyla are reported.

**Phylum**	**CMA**	**CMS**	**HEALTHY**	**p value**
Firmicutes	46.7057	59.8204	28.2082	**0.034**
Verrucomicrobia	1.38533	1.33902	5.82754	**0.00011**

Supplementary Table S6. DeSeq2 test statistically significant results for CMA, CMS and HEALTHY group comparison at genus level. For each group mean relative abundances of genus are reported.

Phylum	Genus	CMA	CMS	HEALTHY	p value
Proteobacteria	*Pasteurellaceae;g_*	0.08007	0.00318	0.02793	**0.01227**
Proteobacteria	*Actinobacillus*	0.26436	0.01586	0.01954	**0.01415**
Proteobacteria	*Haemophilus*	4.33221	0.70116	0.23531	**0.0399**
Proteobacteria	*Erwinia*	1.75906	0.08571	0.19719	**0.01227**
Proteobacteria	*Klebsiella*	6.87882	0.19301	2.65591	**0.0011**
Bacteroidetes	*Prevotella*	0.6829	0.03561	0.04084	**0.01827**
Bacteroidetes	*Parabacteroides*	0.01198	0.07964	0.21774	**0.01827**
Firmicutes	*Granulicatella*	0.02863	0.03899	0.00551	**0.0399**
Firmicutes	*Streptococcus*	2.26979	0.70702	0.28369	**0.01744**
Firmicutes	*Anaerotruncus*	0	0.17253	0.00675	**0.00896**
Firmicutes	*Ruminococcaceae_g_*	1.09775	6.97716	1.94288	**0.01415**

values in bold are statistically significant.

Supplementary Table S 7. DeSeq2 test statistically significant results for T_0, T_1, T_3 and T_5 group comparison at phylum level. For each group mean relative abundances of phyla are reported.

Phylum	T_0	T_1	T_3	T_5	p value
Actinobacteria	27.702	42.6975	46.95328742	32.8538	**0.004816519**
Proteobacteria	31.483	4.32714	11.90355827	0.72093	**0.004934564**
Verrucomicrobia	2.02595	0.85829	4.688481257	0.02854	**9.08×10^{-9}**

values in bold are statistically significant.

Supplementary Table S8. DeSeq2 test statistically significant results for T_0, T_1, T_3 and T_5 group comparison at genus level. For each group mean relative abundances of genus are reported.

Phylum	Genus/Species	T_0	T_1	T_3	T_5	p value
Actinobacteria	*Actinomyces*	0.04224	0.035964078	0.00834	0.00099	**0.004550742**
Verrucomicrobia	*Akkermansia*	0.02134	2.75815	0.16394	16.1942	**0.00317937**
Firmicutes	*Blautia*	1.54716	2.217692621	0.25798	0.24496	**0.033548925**
Firmicutes	*Enterococcus*	0	0.543750424	0.00346	0	**0.023335909**
Bacteroidetes	*Prevotella*	0.01077	0.018769552	0	0.00137	**0.04779255**
Firmicutes	*Ruminococcus*	0.0397	0.006703222	0.00947	0.08133	**0.033548925**
Firmicutes	*Streptococcus*	0.49146	0.848348	0.24725	0.81385	**1.47×10^{-5}**
Proteobacteria	*Sutterella*	3.16174	0.003268989	0	0.01057	**0.033548925**

values in bold are statistically significant.
